# Neurological Phenotypes in Mouse Models of Mitochondrial Disease and Relevance to Human Neuropathology

**DOI:** 10.3390/ijms24119698

**Published:** 2023-06-02

**Authors:** Elizaveta A. Olkhova, Laura A. Smith, Carla Bradshaw, Gráinne S. Gorman, Daniel Erskine, Yi Shiau Ng

**Affiliations:** 1 Wellcome Centre for Mitochondrial Research, Faculty of Medical Sciences, Newcastle University, Newcastle upon Tyne NE2 4HH, UK; elizaveta.olkhova@newcastle.ac.uk (E.A.O.); laura-alexandra.smith@newcastle.ac.uk (L.A.S.); carla.bradshaw@newcastle.ac.uk (C.B.); grainne.gorman@newcastle.ac.uk (G.S.G.); daniel.erskine@newcastle.ac.uk (D.E.); 2 Translational and Clinical Research Institute, Faculty of Medical Sciences, Newcastle University, Newcastle upon Tyne NE2 4HH, UK; 3 NHS Highly Specialised Service for Rare Mitochondrial Disorders, Newcastle upon Tyne Hospitals NHS Foundation Trust, Newcastle upon Tyne NE2 4HH, UK; 4 NIHR Newcastle Biomedical Research Centre, Biomedical Research Building, Campus for Ageing and Vitality, Newcastle upon Tyne NE4 5PL, UK

**Keywords:** cerebellar ataxia, neurodegeneration, inhibitory neurons

## Abstract

Mitochondrial diseases represent the most common inherited neurometabolic disorders, for which no effective therapy currently exists for most patients. The unmet clinical need requires a more comprehensive understanding of the disease mechanisms and the development of reliable and robust in vivo models that accurately recapitulate human disease. This review aims to summarise and discuss various mouse models harbouring transgenic impairments in genes that regulate mitochondrial function, specifically their neurological phenotype and neuropathological features. Ataxia secondary to cerebellar impairment is one of the most prevalent neurological features of mouse models of mitochondrial dysfunction, consistent with the observation that progressive cerebellar ataxia is a common neurological manifestation in patients with mitochondrial disease. The loss of Purkinje neurons is a shared neuropathological finding in human post-mortem tissues and numerous mouse models. However, none of the existing mouse models recapitulate other devastating neurological phenotypes, such as refractory focal seizures and stroke-like episodes seen in patients. Additionally, we discuss the roles of reactive astrogliosis and microglial reactivity, which may be driving the neuropathology in some of the mouse models of mitochondrial dysfunction, as well as mechanisms through which cellular death may occur, beyond apoptosis, in neurons undergoing mitochondrial bioenergy crisis.

## 1. Introduction

Mitochondrial diseases, characterised by a primary perturbation in mitochondrial activity, comprise the largest group of inherited metabolic diseases, affecting approximately 1 in 4300 individuals [1]. Mitochondrial dysfunction may result from either a direct or indirect defect of the oxidative phosphorylation (OXPHOS) system [2] or a deficit in the tricarboxylic acid cycle or fatty acid oxidation [3], resulting in a bioenergy deficit. The mitochondrial OXPHOS system comprises five multi-subunit complexes embedded in the inner mitochondrial membrane and generates the majority of adenosine triphosphate (ATP) molecules per molecule of glucose [4].

The mitochondrion is a unique organelle that harbours its own genome, termed mitochondrial DNA (mtDNA), which encodes 13 essential subunits of the mitochondrial OXPHOS machinery, specifically belonging to complexes I, III, IV, and V. However, the majority of proteins localised to the mitochondria (>95%) are nuclear DNA (nDNA) encoded proteins, which are synthesised in the cytoplasm and subsequently imported into the mitochondrion [5]. Thus, mitochondria are under dual genomic control, and pathogenic variants in any mtDNA or nDNA genes that encode for mitochondrial proteins could theoretically cause mitochondrial disease. This may include pathogenic variants in genes encoding subunits or assembly factors of the OXPHOS system; it may also include proteins required for replication, transcription, or translation of mtDNA, or mitochondrial quality control proteins [3,6,7].

Enormous heterogeneity exists in mitochondrial disease, including high variability at the age of onset, the severity of clinical presentation, and tissue- or organ-specific involvement, even for patients harbouring the same pathogenic variant [2]. For example, bi-allelic pathogenic variants in *POLG*, the gene encoding the catalytic subunit of the mitochondrial DNA polymerase gamma (Polɣ), cause a diverse spectrum of clinical phenotypes ranging from paediatric myocerebrohepatomyopathy spectrum disorder, and Alpers’ syndrome, to adult-onset chronic progressive external ophthalmoplegia (CPEO) and ataxia neuropathy spectrum (ANS) [8,9]. Furthermore, the pathogenic m.3243A > G variant within *MT-TL1*, encoding for mitochondrial tRNA^Leu(UUR)^, may result in maternally inherited diabetes and deafness (MIDD), or severe mitochondrial encephalomyopathy, lactic acidosis, and stroke-like episodes (MELAS syndrome), or non-syndromic multisystem involvement [10,11]. This remarkable variability is thought to arise, at least in part, due to the relative abundance of mutated mtDNA in relation to wild-type mtDNA present within cells and tissues, known as the mtDNA heteroplasmy [12]. However, pathogenic variant heteroplasmy cannot fully explain clinical phenotypes in patients harbouring the m.3243A > G variant [13]; thus, nuclear modifying factors may contribute to the clinical heterogeneity [13,14].

Central and peripheral nervous systems (CNS and PNS, respectively) are commonly affected in patients with mitochondrial disease. Acute paroxysmal neurological manifestations, such as stroke-like episodes, status epilepticus, and brainstem dysfunction, are potentially life-threatening and associated with significant functional impairment [15,16]. On the other hand, patients can develop more chronically progressive neurological impairments, such as ataxia, dementia, dystonia, myopathy, and neuropathy, which are debilitating morbidities that compromise patients’ quality of life [15,16,17,18,19]. The underlying pathomechanisms of these diverse neurological impairments and neurodegenerative processes are yet to be fully elucidated; therefore, the generation of the model systems in which the pathogenesis can be interrogated spatially and temporally is critical to aid the identification of cellular targets and molecular pathways amenable to therapeutic intervention. This requires suitable and robust in vivo models to recapitulate diverse neurological impairments experienced by patients with mitochondrial disease.

Management of primary mitochondrial diseases is extremely challenging due to limited therapeutic strategies that are mainly symptomatic and not curative [20]. The lack of fundamental understanding of disease mechanisms and organ and tissue specificity of mitochondrial dysfunction caused by different genetic defects have been the major barriers to therapeutic discovery in mitochondrial medicine [20].

Neurons are highly metabolically active cells due to the physiological processes underpinning normal neural functions, including the generation of action potentials and maintenance of the ionic resting membrane potential using the Na^+^/K^+^ ATPase pumps embedded in the plasma membrane [21]. The synthesis, exocytosis and recycling of neurotransmitters, and buffering of Ca^2+^ ions are critical for neural activity, in which mitochondria participate as a Ca^2+^ ion buffering organelle in conjunction with the endoplasmic reticulum [22]. However, diverse neuronal subtypes possess variable biophysical and electrophysiological properties and, thus, may differ in their ATP and Ca^2+^ buffering requirements, including the dendritic tree arborisation, length of axons, the rate of action potential generation, and size of the cell body. This may therefore implicate a differential involvement of specific neuronal subtypes in mitochondrial disease where mitochondrial function is impaired; addressing cell-autonomous factors that could mediate such selective vulnerability is important in delineating the pathobiology underlying neurological manifestations of primary mitochondrial diseases.

The selective vulnerability of brain regions in different mitochondrial syndromes and genetic defects exists: the pronounced predilection of stroke-like lesions towards posterior brain regions [16] and basal ganglia and brainstem involvements in Leigh syndrome [23] have been consistently reported in human studies. However, the underlying mechanisms, which could explain specific brain region predisposition to lesion formation in different mitochondrial genetic syndromes, are poorly understood. Therefore, detailed characterisation of neuronal and glial changes in response to mitochondrial dysfunction in different brain regions is crucial for the development of therapeutic strategies to ameliorate neurological impairment in patients with mitochondrial disease. Mammalian in vivo models of mitochondrial disease are invaluable for delineating the complex pathomechanisms underpinning genotype–phenotype relationships and preclinical treatment studies. Unravelling tissue and cellular vulnerability in mitochondrial diseases is feasible using pathogenic global or conditional knockout or knockin genetic methods and applying them to different cell types (for example astrocytes, excitatory neurons, and inhibitory neurons).

This review aims to summarise and discuss current advances in the field of murine models of mitochondrial dysfunction that manifest with a neurological phenotype, focusing on whether certain neuronal or glial populations, as well as brain regions, might show exacerbated susceptibility to mitochondrial dysfunction. Although mouse models do not recapitulate the full spectrum of human mitochondrial disease [24], they are useful in elucidating pathogenic mechanisms. In the scope of this review, we included genetically modified mouse models that harbour knockouts, knockins, or point mutations in nDNA genes that encode subunits or mobile electron carriers of the mitochondrial OXPHOS system; mtDNA maintenance enzymes, including Twinkle helicase (*Twnk*), mitochondrial transcription factor A (*Tfam*), and DNA polymerase gamma (*Polg*); thymidine kinase 2 (*Tk2*); and proteins that regulate mitochondrial dynamics (*Drp1*, *Mfn2*, *Miro1*) and protein import (*Tim23*).

## 2. Mitochondrial Dysfunction in Excitatory Forebrain Neurons Results in Late-Onset Neurodegeneration

Global and conditional knockout mouse models allow investigation of selective vulnerability of neuronal or glial cell types to mitochondrial impairment and/or neurodegeneration in mitochondrial disease (Supplementary Table S1).

A summary of murine models of mitochondrial dysfunction selectively within Ca^2+^/calmodulin-dependent protein kinase II (CaMKII)-positive forebrain neurons, which are typically composed of excitatory cells located in the cerebral cortex and hippocampus, is presented in Table 1.

MtDNA depletion models in CaMKII-expressing cells, including mitochondrial late-onset neurodegeneration (MILON) mice harbouring *Tfam* knockout [25]—Tfam is necessary for the maintenance of mtDNA copy number [26,27] and mtDNA helicase Twinkle-encoding gene *Twnk* knockout [28]—both show late-onset neurodegeneration. These mouse models with a selective excitatory neuron mitochondrial impairment did not display an overt clinical phenotype until immediately prior to their death in both studies at 5–6 months and 7–8 months of age, respectively [25,28]. Neurodegeneration was accompanied by secondary astrogliosis in both models, defined as increased astrocytic glial fibrillary acidic protein (GFAP) expression and increased area of the brain region occupied by GFAP-immunoreactive astrocytes due to their increased cell density and size [25,28].

Another CaMKII-specific in vivo model harbouring a conditional knockout (cKO) of *Drp1*, a gene encoding a key player in mitochondrial fission termed dynamin-related protein 1 (Drp1), exhibited a hippocampus-dependent working memory impairment, altered synaptic neurotransmission in the CA1 region of the hippocampus, and a reduced hippocampal volume. However, no evidence of cell loss was observed despite the hippocampal atrophy [29].

Detailed phenotypic characterisation of a mouse model accumulating mtDNA deletions in forebrain neurons due to a knockin of *Polg* with a deficient proof-reading domain did not reveal any neurological changes [30]. Specifically, there were no perturbations in motor coordination or cognition, nor signs of depression or anxiety [30]. The affected mice had normal life span, and the sole disturbances were documented in the diurnal cycle of the mutated mice by studying the wheel-rotation activity within a 12 h light/dark cycle [30]. Transgenic mice tended to continue the wheel-rotating behaviour after the lights were switched on when mice are typically asleep and immediately prior to the lights being switched off [30]. These phenomena were termed by the authors as delayed activity and anticipatory activity, which indicate an altered diurnal cycle in comparison with the control mice [30].

Complex III and complex IV OXPHOS defects were modelled in mice through the cKO of Rieske-iron sulphur-cluster protein-encoding gene *RISP* (termed *Uqcrfs1* gene, which encodes for a structural subunit of complex III) and *Cox10*, which encodes for an assembly factor of complex IV in CaMKII-positive neurons [31]. Interestingly, it was evident that complex III knockout led to a significantly earlier death at approximately 3 months of age, in contrast to complex IV knockout with a greater median survival of approximately 10 months of age [31]. The early death of *RISP* cKO mice could be associated with an earlier and more severe oxidative stress, which was detected using immunohistochemical methods to label 8-hydroxy-guanosine (a marker of RNA/DNA oxidative damage) and immunoblotting analysis of N-tyrosine and 4-hydroxynonenal (4-HNE) levels, indicative of nitrosative stress and lipid peroxidation, respectively [31]. However, whether the oxidative and nitrosative stress play a causal role in triggering cell loss in this mouse model is unknown.

Another study employed a cKO of the complex I subunit-encoding *Ndufs4* gene specifically within excitatory neurons that express the glutamatergic marker Vglut2. Affected mice had a severely reduced life span and motor impairment with symptoms including tremor, reduced locomotion, ataxia, hypotonia, and breathing difficulties, despite the reported lack of neurodegeneration, or neuronal cell loss [32].

**Table 1:** Summary of mouse models harbouring conditional knockouts in Ca^2+^/calmodulin-dependent kinase II (CaMKII)-expressing neurons.

Publication	Transgenic Mouse Model	Mitochondrial Dysfunction	Onset	Phenotype	Neuropathological Features	Life Span
Sörensen et al., 2001 [25]	*Tfam* knockout (CaMKII-cre) (‘MILON’)	mtDNA depletion; complex I and IV dysfunction.	5 months	Premature death due to deteriorating physical condition.	Axonal and neuronal degeneration, gliosis, TUNEL+ nuclei in the cortex and hippocampus. No changes in cleaved caspases 3 or 7 expressions.	5.5–6 months
Ignatenko et al., 2018 [28]	*Twnk* knockout (CaMKII-cre)	mtDNA depletion from 3–4 months; complex I, II, III, IV downregulation at 7–8 months.	7–8 months	No abnormalities in beam walk or grip strength tests.	GFAP+ astrocyte activation in the cortex at 7–8 months. Some COX-deficient neurons in the cortex and hippocampus at 5–6 months. NeuN and calbindin neuronal densities were preserved. No changes in caspase-3 in the cortex, increase in the hippocampus CA1 region. Increase in FJ-positive cells in the cortex at 7–8 months.	7–8 months
Oettinghaus et al., 2016 [29]	*Drp1* conditional knockout (CaMKII-cre)	Reduction in Drp1 expression; reduced oxygen consumption and ATP production in isolated hippocampal mitochondria.	4 weeks (post-injection)	Hippocampus-dependent working memory impairment at 4 weeks post-knockout.	Impairment in synaptic transmission in CA1. No changes in action potential parameters. Reduced number of presynaptic terminals containing mitochondria. Decreased hippocampal volume 10 weeks post-knockout. No neurodegeneration and no differences in TUNEL-positive cell density.	10 weeks post-injection (no overt phenotype)
Kasahara et al., 2006 [30]	*Polg* p.D181A knockin (CaMKII-cre)	mtDNA deletion in brain tissue.	21–28 weeks	Reduced body weight. Enhanced acoustic startle response. Diurnal rhythmicity perturbation. No anxiety, locomotion, or cognitive impairment.	DA and 5-HT content and turnover were altered in the amygdala and hippocampus.	Unaffected
Diaz et al., 2012 [31]	*Uqcrfs1* (RISP) and *Cox10* (CaMKII-cre)	RISP: Reduction in complex III. Cox10: Reduction in complex IV.	RISP: 2 months COX10: 3 months	Reduced body weight. RISP: coordination impairment. COX10: alternate cycles of hyperactivity and movement impairment.	RISP: lesion in the piriform cortex, similar to COX10, in which it was followed by striatal, cortical, and hippocampal lesions. Increased markers of oxidative stress in both models. TUNEL staining showed positive neurons with neuronal loss detected in COX10 cKO; astrogliosis.	RISP: 3–3.5 months COX10: 8–12 months

5-HT—5-hydroxytryptamine; ATP—adenosine triphosphate; CA—cornu ammonis; Cox10—cytochrome c oxidase subunit 10; DA—dopamine; Drp1—dynein-related protein 1; FJ—Fluoro-Jade assay; GFAP—glial fibrillary acidic protein; Polg—polymerase gamma; RISP—Rieske iron-sulphur protein; Tfam—mitochondrial transcription factor A; TUNEL—Terminaldeoxynucl eotidyl transferase dUTP nick end labelling; Twnk—Twinkle (mtDNA helicase); Uqcrfs1—ubiquinol cytochrome c reductase.

## 3. Vulnerability of the Cerebellum to Neuropathological Changes in Global Transgenic In Vivo Models

Global or Nestin-specific (denotes neural stem cells or neural progenitor cells) pathogenic knockouts or knockins of various genes involved in maintaining mitochondrial function have interestingly reported discrete brain regions to be affected, with most models consistently showing a strong predilection for cerebellar involvement (Supplementary Table S1).

Severe ataxia is one of the most common neurological features recapitulated by mouse models of mitochondrial dysfunction due to various genetic defects (summarised in Table 2). This includes mouse models of complex I deficiency due to the knockout of *Ndufs4*; models of mtDNA depletion syndromes due to *Tk2* knockout or *Twnk* mutations; models of accumulating mtDNA point mutations due to *Polg* pathogenic variants (e.g., “mutator” mouse model); and models due to mitochondrial protein import impairment in heterozygous *Tim23* knockout mice [33,34,35,36,37,38,39]. The motor phenotype displayed by the aforementioned mouse models of mitochondrial disease was commonly characterised by reduced motor coordination assessed by the rotarod or beam walk tests, gait instability, or an overall decrease in locomotion in the open field.

Neuropathological studies of harvested mouse brain tissues corroborate phenotypic data. Mouse models of complex I deficiency, coenzyme Q10 disorder, and mitochondrial import defects demonstrated spongiotic encephalopathy in the cerebellum [34,39,40]. Additional studies investigating models of impaired mtDNA replication and depletion provided evidence for the selective degeneration of inhibitory Purkinje cells [35,41] (Table 2). Cerebellar Purkinje cells provide the sole output from the cerebellar cortex, controlling the activity of the deep cerebellar nuclei via GABAergic neurotransmission [42]. Neuropathological studies using post-mortem brain tissues from patients with mitochondrial disease demonstrate a severe degeneration of Purkinje cells and OXPHOS deficiencies in those remaining [17,43,44]. The dentate nucleus of the deep cerebellar nuclei is also frequently affected by neurodegeneration in tissues from patients with mitochondrial disease [43,45]; however, specific changes to the deep cerebellar nuclei are rarely reported in brain tissues derived from murine models of mitochondrial disease. Degeneration of Purkinje cells and neurons of the dentate nucleus is hypothesised to underpin cerebellar ataxic symptoms, which comprise one of the most commonly observed neurological features, with the prevalence of approximately 60% in adult and 50% in paediatric patient populations with mitochondrial disease, respectively [46,47].

The predilection of Purkinje cells to neurodegeneration and the profound level of OXPHOS deficiencies observed within surviving Purkinje cells in mitochondrial disease [17,43,44] could be explained in part by their size and patterns of neuronal activity they can generate. Purkinje neurons are one of the largest neurons of the brain, and due to the greater cell surface area they possess compared with other neuronal classes, a higher degree of dependence on ATP molecules is required to sustain their resting membrane potential (via Na^+^/K^+^ ATPases) and handling of larger ionic fluxes [48]. Purkinje cells have a unique pacemaking activity with high intrinsic variability in firing frequency ranging between 5 and 80 Hz [49]. Additionally, frequency rates of 200 Hz or above in simple spikes were described in acute ex vivo cerebellar slices from mice [50]. Purkinje cells also produce complex spikes, which are short bursts with a high-frequency spikelet component of up to 600 Hz, in response to strong excitatory synaptic input from the climbing fibres originating in the inferior olivary nucleus [51]. Inferior olivary nucleus neurons are also severely affected in mitochondrial disease [43]. Moreover, millisecond-precise synchronisation of Purkinje cells within the Purkinje cellular layer is documented, with oscillations reaching high frequencies in the range of 150–250 Hz in vitro and in vivo [52], which are thought to underlie learning and control of coordinated movements [53]. Thus, the unique electrophysiological properties of inhibitory Purkinje cells may render them particularly vulnerable to dysfunction and degeneration in the presence of mitochondrial impairments, which may explain the common pathological involvement of these cells in patients with mitochondrial disease and in murine in vivo models of mitochondrial dysfunction. Corroborating this, patch clamp recordings in *Mfn2*-knockout Purkinje neurons revealed that mitochondrial dysfunction non-significantly reduced the action potential generation rate of the surviving cells [54].

**Table 2:** Summary of mouse models displaying features of cerebellar ataxia.

Publication	Transgenic Mouse Model	Mitochondrial Dysfunction	Onset	Phenotype	Neuropathological Features	Life Span
Kruse et al., 2008 [33]; Quintana et al., 2010 [34]; McElroy et al., 2020 [55]; Lee et al., 2019 [56]	*Ndufs4* global or Nestin-specific conditional knockout	complex I knockout	3 weeks	Weight loss, ataxia, lethargy, loss of motor coordination (rotarod), blindness, cataracts, visual cliff test failure, enhanced startle response, hindlimb clasping, lower body temperature, and reduced locomotion in the open field.	Spongiform degeneration in the olfactory bulb, cerebellum, and vestibular nuclei. Vascular proliferation in brainstem and midbrain. Activation of caspase-8, no changes in cleaved caspase-3. Necrotic cell death, infiltration of phagocytic microglia. Purkinje cell neurodegeneration. Reduced NAD+.	7 weeks
Bolea et al., 2019 [32]	*Ndufs4* conditional knockout in Vglut2-expressing neurons	complex I knockout	40 days	Reduced life span, weight, and body temperature. Tremor, motor coordination impairment (rotarod), ataxia, hindlimb clasping, hypotonia, decreased movement (open field), and breathing difficulties.	Lack of neuronal loss. Reduced activity of neurons in vivo. Increased caspase-8 activation in the vestibular nucleus. Increased gliosis in the vestibular nucleus, inferior olive, and fastigial nucleus.	67 days
Akman et al., 2008 [57]; Siegmund et al., 2017 [36]	*Tk2* H126N global knockin	mtDNA depletion disorder	7 days	Weakness, reduced body weight, reduced movement in the open field test, rotarod impairment.	Encephalomyelopathy, astrocytic proliferation.	15 days
Bartesaghi et al., 2010 [35]	*Tk2* global knockout	mtDNA depletion disorder	7 days	Ataxia and gait abnormalities.	Purkinje cell neurodegeneration. No TUNEL staining in Purkinje neurons, only in granule cells of the cerebellum.	12 days
Song et al., 2012 [38]	*Twnk* 353-365 duplication overexpression (global knockin)	mtDNA deletions accumulation		No weight changes. Rotarod dysfunction at 23 months of age.	Neuronal loss in substantia nigra at 16–23 months of age. No evidence of apoptosis via the TUNEL assay.	No life span changes
Dai et al., 2014 [37]	*Polg* global D257A knockin (‘Mutator’)	mtDNA somatic mutation accumulation	12–14 months	Reduced body weight, locomotion, and rotarod coordination impairment.	Reduction of tyrosine hydroxylase immunoreactivity in substantia nigra, without neurodegeneration.	No life span changes
Ahting et al., 2009 [39]	*Tim23* global heterozygous knockout	Mitochondrial protein import dysfunction	4 months	Reduced grip strength in female mice. Rotarod motor coordination impairment. No hearing or visual impairments.	N/A	9 months
Chen et al., 2007 [58]	*Mfn2* knockout from cerebellar primordial cells	Mitochondrial morphology impairment	1 day	Motor imbalance, poor feeding	Progressive Purkinje neuron loss, granule cells unaffected. TUNEL+ staining.	15 days

Ndufs4—NADH:ubiquinone oxidoreductase subunit S4; Polg—polymerase gamma; Tim 23—translocaase of inner mitochondrial membrane 23; Tk2—thymidine kinase 2; Twnk—Twinkle (mtDNA helicase); Vglut2—vesicular glutamate transporter (solute carrier family 17 member 6).

## 4. Susceptibility of Pacemaking Activity-Generating Neurons in Mitochondrial Disease Models

Neurons exhibiting a similar pattern of spontaneous pacemaking activity to that of Purkinje cells also include neurons found in the basal ganglia and midbrain, which are also commonly affected in mitochondrial disease. For instance, in POLG-related diseases, some adult patients develop parkinsonism with abnormal dopamine transporter scans, and the neuropathological studies have revealed high heteroplasmy levels of mtDNA deletions and depletion in substantia nigra neurons of the midbrain and severe nigrostriatal neurodegeneration [59,60,61,62]. Moreover, symmetrical MRI signal abnormalities involving the basal ganglia constitute the most common radiological finding in Leigh syndrome [63], followed by hyperintensities within the substantia nigra [64].

To decipher the role of mitochondrial defects in the aetiology of parkinsonism and movement disorders, mtDNA depletion was modelled using a conditional knockout of *Tfam* in dopamine transporter (DAT)-expressing neurons, which are present in the substantia nigra [65]. This model is termed the MitoPark mice, as they show cardinal features of Parkinson’s disease (PD), including an adult-onset reduction in movement with a progressive disease course, selective neurodegeneration of the substantia nigra rather than ventral tegmental area (VTA), which also contains a high density of dopaminergic neurons, and responsiveness to the administration of levodopa dopaminergic precursor therapy widely used in the motor symptom management of PD [65,66].

A later study intriguingly demonstrated that cognitive impairment in spatial and recognition memory, assessed via the Barnes maze and novel object recognition (NOR) tests, respectively, preceded the emergence of a motor phenotype of MitoPark mice by four weeks [67], highlighting the need for routine cognitive behavioural testing of murine models of mitochondrial disease. The Barnes maze and NOR tests employed by the authors of the study are dependent on distinct brain regions, namely, the hippocampus, prefrontal and perirhinal cortex, and entorhinal cortex, respectively, implicating a widespread cognitive dysfunction that affects different brain regions. A more recent study by Langley and colleagues demonstrated that MitoPark mice exhibited anxiety-like phenotype and behavioural despair at 14 weeks of age, as well as spatial cognitive deficits in the Morris water maze at 24 weeks of age [68]. Interestingly, the same study demonstrated a decrease in the expression of brain-derived neurotrophic factor (BDNF) in the hippocampus, despite its concomitant increase in the striatum [68], which could in part explain spatial memory deficits observed in this murine model [69]. Cognitive impairment alongside other neuropsychiatric symptoms has been documented in PD and prodromal PD prior to the onset of motor symptoms [70].

Cognitive deficits are observed in over 40% of adult patients with mitochondrial disease [71]. A systematic review concluded that there is a highly varied prevalence of cognitive decline and dementia in various forms of mitochondrial disease, which requires further investigation [71]. The exact pathomechanisms of cognitive impairment in the context of primary mitochondrial diseases have not been elucidated; therefore, the generation of robust animal models that recapitulate this phenotypic feature is crucial.

## 5. Mitochondrial Dysfunction in Inhibitory Neurons and Its Implications in Epilepsy

Approximately half of children and a quarter of adult patients with mitochondrial disease experience epilepsy [18,72,73]; however, mouse models that faithfully recapitulate this phenotype in the context of primary mitochondrial dysfunction are extremely scarce. While focal epilepsy is the most common type of epilepsy observed in patients with mitochondrial disease, some mouse models of mitochondrial dysfunction display signs of generalised epilepsy, rather than focal seizures [32,34,41].

Infantile-onset spinocerebellar ataxia (IOSCA) is a neurological disorder of infancy that develops after one year of age with ataxia, hypotonia, followed by ophthalmoplegia, optic atrophy, and sensorineural deafness by seven years of age [74]. Interestingly, an IOSCA mouse model with a global Y509C knockin mutation within the *Twnk* gene demonstrated adult-onset seizures at 6 months of age in 5 of 21 genetically modified male animals, despite limited evidence of significant mtDNA depletion levels in the brain [41]. However, the IOSCA transgenic model lacked any concomitant motor disturbances, and, therefore, did not recapitulate key phenotypic features of IOSCA disorder characterised primarily by severe ataxia and hypotonia [41].

A global and Nestin-specific conditional knockout of complex I subunit *Ndufs4* in mice, modelling key features of Leigh syndrome, resulted in early-onset tonic-clonic seizures in a proportion of animals [34,55,75]. However, *Ndufs4* gene therapy was able to eliminate tonic-clonic seizures, which were observed in 23% of the mice that were administered a control vector (vehicle-treated). *Ndufs4* gene therapy also improved complex I activity, reduced Purkinje cell loss, ameliorated the neurological phenotype (clasping), increased locomotion and reduced rotarod deficits, and extended the life span more than fourfold compared with vehicle-treated mice [75]. Another study replicated these data independently, showing that a single injection of a viral vector containing the *Ndufs4* gene is sufficient to restore complex I expression and activity, improve motor phenotype, reduce gliosis in the vestibular nucleus, and improve body weight and life span [76].

It remains challenging to dissect the involvement of specific neuronal subtypes in the emergence of epileptogenesis in genetically modified models that harbour global impairment; thus, models that display mitochondrial dysfunction within specific neuronal or glial subtypes are required. A publication by Bolea et al. (2019) compared three cKO mouse models harbouring *Ndufs4* deletion in different neuronal subtypes [32]. Selective knockout of complex I specifically within GABAergic inhibitory cells was sufficient to cause the development of early-onset tonic-clonic seizures in mice [32], highlighting the importance of intact mitochondrial function within the inhibitory neurons, which are critical for the regulation of neuronal networks. Importantly, the authors showed that the anti-epileptic drugs levetiracetam, perampanel, or carbamazepine failed to exert any therapeutic effect and did not significantly suppress the frequency of seizures in the GABAergic *Ndufs4^−/−^* mice, recapitulating the refractory nature of mitochondrial epilepsy [32].

Drug-resistant epilepsy remains one of the major challenges in the management of mitochondrial disease, especially in childhood-onset POLG-related diseases, such as Alpers’ syndrome, where refractory status epilepticus are the predominant neurological manifestation [77,78]. The findings from the *Ndufs4* GABAergic knockout mice implicating a causal pathophysiological role of inhibitory neurons in mitochondrial epilepsy are consistent with previous reports of reduced inhibitory neuron densities and decreased complex I and IV expression profiles within the remaining GABAergic cells in post-mortem brain tissues from patients with mitochondrial disease [44,79,80]. Moreover, human neuropathological studies have demonstrated a decreased vulnerability of pyramidal excitatory neurons to neurodegeneration and mitochondrial OXPHOS protein deficiencies [44], indicating the potential existence of selective inhibitory neuronal susceptibility, which is hypothesised to result in network disinhibition and precipitation of seizure activity [81].

## 6. Parvalbumin-Expressing Neurons Demonstrate Vulnerability in Mitochondrial Disease

Mouse models with a transgenic defect resulting in mitochondrial dysfunction selectively in CaMKII-positive excitatory cells lack seizure activity, despite evidence of neurodegeneration of these neurons [25,28], indicating the involvement of other distinct neuronal subtypes in the pathogenesis of mitochondrial epilepsy. For example, current evidence suggests that the inhibitory neurons could be driving some important disease phenotypes [32].

**Figure 1:**
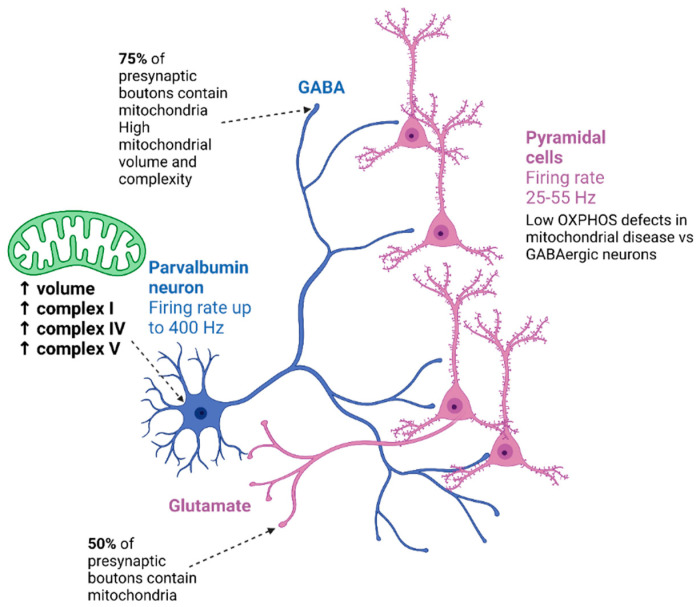
Comparison of excitatory pyramidal and PV+ inhibitory neuronal mitochondrial content and firing rates. Based on data described in [44,82,86,87,88,89,90,91]. Diagram was created using biorender.com.

Certain inhibitory neuronal subclasses are hypothesised to be particularly vulnerable to mitochondrial dysfunction, with parvalbumin-expressing (PV^+^) neurons suggested to be preferentially affected due to their high frequency of firing and lack of accommodation (Figure 1) [82]. PV^+^ interneurons constitute approximately 40% of all inhibitory GABAergic neurons in the cortex [83], with the majority displaying fast-spiking properties, which renders them vulnerable to bioenergy deficits [82] as evidenced by mitochondrial OXPHOS inhibitors blocking their fast-spiking action potential generation capacity [84]. Indeed, a recent study has comprehensively demonstrated severe neurodegeneration and deficiencies of mitochondrial OXPHOS components in PV^+^ cortical interneurons compared with calretinin-expressing (CR^+^) interneurons in post-mortem brain tissues from patients with Alpers’ syndrome [80]. Moreover, the same neuropathological study demonstrated that CR^+^ interneurons appear to be more resilient to neurodegeneration compared with PV^+^ and calbindin-expressing (CB^+^) interneurons, even within focal cortical necrotic lesions in some patient tissues [80]. In addition, it was reported that in MELAS syndrome, a reported loss of CB^+^ expression in the hippocampus from patients harbouring the m.3243A > G variant, was associated with cognitive impairment [85]. However, further post-mortem studies are required to dissect the individual inhibitory neuron subclass vulnerability to mitochondrial dysfunction and their contribution to neurological deficits in patients with mitochondrial disease.

A study by Inan and colleagues explored the consequences of complex IV downregulation specifically within PV^+^ neurons using a cKO of *Cox10* in mice [92]. Importantly, Inan et al. demonstrated that in control animals Cox1, the catalytic subunit of complex IV is differentially expressed in interneurons of the somatosensory cortex [92]. PV^+^ interneurons exhibited the highest Cox1 expression levels, measured by the percentage area stained within individual interneurons compared with somatostatin-expressing (SST^+^) and CR^+^ interneurons [92]. These findings further implicate higher metabolic demand of PV^+^ interneurons compared with other inhibitory subclasses. The mice with a *Cox10* cKO in PV^+^ neurons demonstrated a schizophrenic-like phenotype with reduced sociability, impaired pre-pulse inhibition, and aberrantly high power of gamma-frequency oscillations in the cortex and hippocampus in vivo [92]. These features are hypothesised to be due to neuronal hyperexcitability, as interneurons have demonstrated a significantly higher rate of spontaneous action potential firing ex vivo [92]. However surprisingly, progressive loss of Cox1 in PV^+^ cells did not cause neurodegeneration in the affected animals; hence, the symptoms mice exhibited were considered to be due to the functional abnormalities of PV^+^ interneurons, rather than their attrition [92]. Since PV^+^ interneurons provide fast inhibition to pyramidal cells in the cortex, their dysfunction has been implicated in many animal models of epilepsy [93]. Despite this, the study did not show evidence of the mice experiencing seizures or movement disorders.

## 7. Mechanisms of Cellular Death in Mitochondrial Disease

Severe forms of mitochondrial disease are characterised by neurodegeneration, which may encompass diffuse neuronal dropout, focal necrotic lesions, or mild neuronal loss. Despite the neurodegenerative pathological findings described in patient tissues, the precise molecular mechanisms and cell death pathways have not been established.

Typically, neuronal or glial cells of the central nervous system (CNS) that are undergoing degeneration can be stained and identified using a Fluoro-Jade fluorescence assay, which utilises a fluorochrome that binds to damaged neurons [94]. However, this assay does not provide information regarding the mode of cellular death and yields a positive signal independently of the specific cell death pathway.

Cell death pathways, such as apoptosis, can be determined via terminal deoxynucleotidyl transferase biotin-dUTP nick end labelling (TUNEL) that identifies cells with fragmented DNA as a result of apoptosis. However, the interpretation of TUNEL staining becomes ambiguous where caspase levels (e.g., cleaved caspase-3, a marker of apoptosis) are unaltered and, hence, do not match the conclusions of the TUNEL findings.

In this review, we summarise some of the mouse model studies that published TUNEL data, with a few of those studies reporting negative findings for TUNEL staining, indicative of the lack of DNA fragmentation and apoptosis [29,38,95] and the remainder describing positive signals detected in neurons in various brain regions, including the hippocampus, cortex, and cerebellum [25,31,35,40,96] (Supplementary Table S1). In one of the studies [35], TUNEL-positive signals were detected in granule cells, which are the excitatory cells of the cerebellar cortex, and not in Purkinje neurons, which were most frequently affected by neurodegeneration. Some studies failed to demonstrate a concomitant increase in caspase-3 levels immunohistochemically [25,40], and in the remaining reports, caspase levels were not determined [31,35,96], which hinders the identification of a specific cellular death mode. Moreover, positive TUNEL staining in the cerebral cortex and hippocampus did not correspond to any changes in pro-apoptotic Bax or anti-apoptotic Bcl-xL transcript levels in MILON mice [25]. Based on TUNEL and caspase activation data, the role of apoptosis within the mouse models of mitochondrial diseases remains inconclusive. One of the reasons for the hampered interpretation of TUNEL results could be confounding necrotic cell death [25] with cellular debris trapping nonspecific chromogenic or fluorescent agents, resulting in false-positive results [97].

Other studies included in this review have investigated caspase-8 and caspase-3 expression levels. There was an increase in caspase-8 expression in a glutamatergic model of *Ndufs4* knockout [32], whereas a global *Ndufs4* knockout showed an increase in caspase-8 levels without any activation of downstream caspase-3 [34]. Quintana and colleagues concluded that ultrastructural changes were indicative of necrotic cell death rather than apoptosis, which was caused by the global complex I deficiency [34].

The cKO of *Twnk* helicase in forebrain neurons showed an increase in cleaved caspase-3 levels at the end-stage of the disease selectively within the CA1 subregion of the hippocampus [28]. Interestingly, cortical regions remained unaffected. Contrasting with the neuronal *Twnk* cKO mice, no changes to caspase-3 expression were detected in the astrocytic *Twnk* cKO mice [28]. Furthermore, in a mouse model of Purkinje neuronal mitochondrial dysfunction via a conditional *Mfn2* knockout, which causes mitochondrial dynamics impairment by blocking fusion, despite the presence of positive TUNEL staining [58], proteomic analysis in a later study revealed a lack of upregulation of apoptosis-related genes [54].

The evidence supporting the role of apoptotic neuronal cell death in post-mortem brain tissues from patients with mitochondrial disease remains scarce; rather, diffuse neuronal dropout and/or laminar cortical necrotic lesions in the neocortex and cerebellum are identified as a neuropathological endpoint and are frequently associated with stroke-like lesions [16,43,98]. Acute necrotic regions correspond to the areas of hyperintensities identified on T2-weighted MRI scans; they are incongruent with vascular territories and are hypothesised to be associated with seizures rather than ischaemia [16,99]. A new consensus-based definition states that the ‘stroke-like episodes’ are paroxysmal events of subacute epileptic encephalopathy [19].

Alternative modes of neuronal cell death, such as phagocytosis by reactive microglia and necroptosis, have not been extensively investigated in post-mortem brain tissues from patients with mitochondrial disease, despite mitochondria playing a central role in regulating alternative pathways of cell death apart from the intrinsic mode of apoptosis. For instance, mitochondrial outer membrane permeabilisation (MOMP) is involved in pro-inflammatory signalling [100]. Furthermore, necroptosis is a mechanism of cell death described as controlled, or programmed, necrosis that is mediated by receptor-interacting serine/threonine kinase 3 (RIPK3) activation, which phosphorylates its substrate mixed-lineage kinase domain-like (MLKL), accompanied by microglial activation and enhanced inflammation [101]. To the best of our knowledge, the examination of this pathway has not been performed in primary mitochondrial disease, but neuronal necroptosis pathway activation is evident in post-mortem reports of Alzheimer’s disease and amyotrophic lateral sclerosis patients [102,103]. The involvement of pro-inflammatory cytokines, such as tumour necrosis factor-alpha (TNF-α), in the induction of necroptosis has been documented [100,104]. Mitochondrial reactive oxygen species (ROS) production is associated with RIPK1 autophosphorylation, which could result in RIPK3 activation and subsequent necrosome formation [105].

Mouse models of parkinsonism, induced by pharmacological complex I inhibition via an injection of 1-methyl-4-phenyl-1,2,3,6-tetrahydropyridine (MPTP), exhibited increased levels of necroptotic markers in the dopaminergic neurons of the substantia nigra [106]. Authors additionally demonstrated that by knocking out necroptotic factors RIPK3 and MLKL it was possible to partially protect substantia nigra neurons from neurodegeneration [106]. A second study demonstrated a partial neuroprotective effect of selective necroptosis inhibitor necrostatin-1 in nigrostriatal areas in the same experimental mouse model of parkinsonism [107].

An alternative pathway of cellular dysfunction in the context of the absence of neuronal loss could be the cellular senescence of neurons, as a result of mitochondrial impairment and inflammation. Mitochondria are essential for senescence to occur [108]; hence, senescence represents an area of interest but surprisingly has not been extensively investigated in the context of mitochondrial disease in post-mortem human brain tissues or in vivo models. Mitochondrial dysfunction and OXPHOS deficiencies constitute a hallmark of cellular senescence with a pro-inflammatory phenotype, accompanied by genomic and proteomic instability caused by elevated ROS levels with activation of two main senescence execution proteins, p21 and p16 [109]. Accumulating oxidative damage and senescence in aged mice was shown in postmitotic cortical neurons and Purkinje cells by Jurk and colleagues [110].

It would be beneficial to determine relevant signalling cascades resulting in neuronal dysfunction and cell death in mitochondrial disease in order to identify specific pharmacological agents that could delay the phenotypic onset or progression by modulating or inhibiting the signalling pathways involved, such as necroptosis inhibitors or senolytic drugs.

## 8. Glial Reactivity in Mitochondrial Disease

Astrocytes are glial cells that have vital roles in supporting blood-brain barrier (BBB) integrity, neurotransmitter synthesis and reuptake, as well as maintaining ion and water homeostasis and supporting and regulating synaptic neurotransmission. Astrocytes have also been shown to take up mitochondria from neighbouring damaged neurons [111]. Although astrocytes are considered to be primarily glycolytic, mitochondrial impairment within astrocytes specifically has not been extensively investigated.

A recent study by Ignatenko et al. (2018) interrogated a mouse model of mtDNA depletion specifically within astrocytes through the cKO of *Twnk* [28]. This study highlighted the contribution of astrocytic mitochondrial dysfunction to spongiotic encephalopathy neuropathological changes in the brain of affected mice. These findings, including spongiform changes, resembled post-mortem neuropathological features reported in brain tissues of patients with Alpers’ syndrome [77]. Astrocytes also adopted a reactive phenotype with an upregulation of glial fibrillary acidic protein (GFAP), which is commonly observed in post-mortem brain tissues from patients with mitochondrial disease [112]. Interestingly, astrocytes also demonstrated a reactive phenotype in the CaMKII neuronal-specific *Twnk* cKO mice [28].

Another murine model of mtDNA depletion specifically within astrocytes introduced a *cre-loxP*-mediated knockout of *Tfam* within glutamate aspartate transporter (GLAST)-expressing astrocytes, which resulted in abnormal mitochondrial swelling within astrocytes and development of a reactive phenotype in the cortex via GFAP upregulation [113]. Following experimentally induced ischaemic strokes, the astrocytic *Tfam* cKO mice showed exacerbated neuronal cell loss and increased levels of cleaved caspase-3 in the perilesional area and reduced astrocytic proliferation, necessary for resolving ischaemic injury [113]. This suggests astrocytic mitochondrial dysfunction is associated with an aberrant response of astrocytes to neuronal insults and a reduced ability to exert the necessary neuroprotection, which may therefore implicate the crucial role of astrocytes in the context of mitochondrial disease.

Similarly to the mtDNA depletion models, *RISP* and *Cox10* cKO models in forebrain neurons of mice demonstrated strong GFAP^+^ astrogliosis in the cortex and hippocampus [25,31]. A mouse model of mtDNA depletion due to a global mutated *Tk2* knockin also resulted in astrocytic reactivity in the brain and spinal cord [57]. Moreover, a global R239X knockin model in the *Coq9* gene, encoding coenzyme Q, also resulted in astrogliosis within the medulla oblongata, pons, and cerebellum [40].

Astrogliosis is also a common finding in post-mortem brain tissues from adult patients with mitochondrial disease and includes prominent astrocytic reactivity observed in proximity to necrotic foci, with astrocytes displaying mitochondrial OXPHOS protein deficiencies [112]. Astrocytes from patients with mitochondrial disease also display decreased expression of glutamine synthetase, which is hypothesised to reflect impaired glutamate metabolism, which, in turn, may exacerbate neuronal hyperexcitability and excitotoxicity [114]. Further interrogation of astrocytic markers that have been implicated in neuronal excitotoxicity and epilepsy, such as aquaporin-4 (AQP4) and inwardly rectifying potassium Kir4.1 channels [115], in patients with mitochondrial disease would better inform the glial pathological changes that could be involved in seizure-associated activity and neurodegeneration in mitochondrial epilepsy.

Microglia, a second type of glial cell, constitute a highly specialised cell type within the CNS that derive from the mesoderm, a distinct lineage to neurons, oligodendrocytes, and astrocytes that originate from the neuroectoderm during embryonic development [116]. Microglia are commonly referred to as the resident macrophages of the CNS, with a plethora of functions, including phagocytosis of pathogens, pruning of synapses during development, as well as phagocytosis of the abnormal degenerating synapses, conferring neuroprotection [117].

Microglial cells become aberrantly activated in numerous pathological states, such as infection or neurodegeneration in Alzheimer’s disease, promoting neuroinflammation and neurotoxicity [117,118]. However, the reactivity, phenotype, and downstream effects of microglial activation have not been well documented in mitochondrial disease post-mortem neuropathological studies, despite the accumulating evidence from mouse models of mitochondrial dysfunction suggesting that extensive microgliosis in affected brain regions is frequently associated with the enhanced phagocytotic function of microglia, and thus could contribute to neuronal loss [118].

Neuropathological studies have demonstrated a high degree of immunoreactive CD68^+^ microglia/macrophages in disease-relevant, selective midbrain regions of post-mortem brain tissues from a patient with Kearns-Sayre syndrome harbouring a large-scale mtDNA deletion [45]. Diffuse microglial activation was also reported in focal necrotic cortical lesions in patients harbouring biallelic pathogenic variants in *POLG* [98]. This may further implicate microglia and/or infiltrating macrophages in the pathogenesis of mitochondrial disease and highlight the importance of further detailed investigation of the role of these cells in human tissues.

In a recent study, it was demonstrated that mitochondrial fragmentation induced selectively within microglia led to an extracellular release of mitochondria, which was sufficient to cause secondary astrocytic activation in vitro and subsequently led to a loss of naive neurons in different mouse models of neurodegeneration [119]. Microglial activation was also evident in a mouse model of *Ndufs4* global knockout [34], and this reactivity was dampened by rapamycin treatment via mechanistic/mammalian target of rapamycin (mTOR) inhibition [120]. Interestingly, both neuronal and astrocytic mouse models of mtDNA depletion due to cKO of *Twnk* showed a secondary change in the morphology of microglia/macrophages, identified by ionised calcium-binding adapter molecule 1 (Iba-1) immunofluorescence, which does not differentiate between macrophages and microglia and therefore may label both cell types [28]. Microglia/macrophages shifted from a ramified to a more amoeboid reactive form with shortened processes despite the lack of change in the overall microglial density [28]. Morphological changes were most pronounced in the end-stage neuronal *Twnk* cKO model, indicating that mitochondrial dysfunction in neurons is sufficient to cause a secondary microglial phenotypic shift. A relatively recent study in MitoPark mice showed a significant upregulation of multiple inflammatory markers in the substantia nigra of mice. This included gp91phox (Nox2), which is an NADPH oxidase subunit required for the production of superoxide radicals by macrophages, inducible nitric oxide synthetase (iNOS), which plays a role in immune system defense, and CD68, suggestive of an inflammatory response [121]. Microgliosis was also evident in the mouse model of *Coq9* R239X knockin and was accompanied by increased expression of pro-inflammatory mediators interleukin 8 (IL-8) and NFκB [122].

Importantly, in a recent study, Aguilar and colleagues demonstrated that depleting microglia and macrophages via colony-stimulating factor receptor 1 (CSF1R) inhibition in a Leigh syndrome *Ndufs4* knockout mouse model prolonged its life span, delayed the onset of clasping, and increased motor coordination in a rotarod test [123]. Interestingly, the neuropathological analysis revealed that depletion of microglia led to a reduction in astrogliosis in the cerebellum but, paradoxically, increased the astrocytic cell densities in the olfactory bulb, vestibular nucleus, cortex, and hippocampus, which reduced neurodegeneration in the olfactory bulb and vestibular nucleus [123]. In contrast, a similar preclinical study carried out by Stokes and colleagues has demonstrated that leucocyte depletion not only resolved necrotic lesions but also abolished Iba-1 reactivity outside of lesions, which was additionally accompanied by decreased GFAP-immunoreactive astrogliosis in the brainstem, cerebellar peduncle, and cortex [124]. In this study, CSF1R blockade had a more profound effect than rapamycin on suppressing inflammation; it also increased *Ndufs4* knockout weight, dramatically reduced the incidence of seizures, ataxia, circling, clasping neurological phenotypes in a dose-dependent manner, and improved breathing in this mouse model [124].

## 9. Mitochondrial DNA Mouse Models Lack a Neurological Phenotype

Approximately 80% of adult patients with mitochondrial disease are carriers of a mtDNA pathogenic variant [1]. Patients harbouring pathogenic mtDNA variants such as m.3243A > G (*tRNA^Leu(UUR)^* gene) and m.8344A > G (*tRNA^Lys^* gene) may present with MELAS and myoclonic epilepsy with ragged-red fibre (MERRF) syndromes, respectively, characterised by neurological diseases, such as ataxia, epilepsy, debilitating stroke-like episodes, and cognitive impairment [16,18]. Despite these syndromes and genetic defects being well described in the literature for a long time, in vivo models recapitulating the neurological phenotypes of MELAS and MERRF syndromes are still lacking. The mtDNA *tRNA^Ala^* gene 5024C > T variant-harbouring high heteroplasmic mouse model exhibited mild cardiomyopathy phenotype and COX deficiencies in smooth muscles and epithelial cells of the colonic crypts [125]. Similarly, the mtDNA *tRNA^Lys^* gene 7731G > A variant mice with high heteroplasmy (over 85%) accumulate age-dependent ragged-red fibres in skeletal muscle and show reduced body weight and grip strength; however, no neurological phenotype was reported [126,127]. The mouse model harbouring *mt-COI* 6589T > C pathogenic variant did not show a neurological phenotype despite reduced complex IV activity in the brain [128]. Furthermore, a recent study by Tani and colleagues generated a mouse model of the *tRNA^Leu(UUR)^* pathogenic variant with affected mice harbouring complex I and complex IV biochemical defects in the brain; however, no neurological phenotype or pathological alterations were reported [129]. Taken together, these reports indicate no neurological phenotype present in the mouse models harbouring mtDNA pathogenic variants.

## 10. Concluding Remarks

In this review, we have discussed a wide range of in vivo mitochondrial disease models that recapitulate neurological phenotypes associated with mitochondrial dysfunction. Importantly, the mouse models have shown the differential impact of selective mitochondrial dysfunction in various cell types that can lead to vastly different phenotypes and neuropathological changes. The literature summarised in this review suggests that mouse models characterised by mitochondrial dysfunction in forebrain excitatory neurons do not tend to exhibit a prominent disease phenotype and show late-onset neuronal death, which occurs immediately prior to death. On the other hand, mitochondrial defect-harbouring inhibitory neurons, such as gamma-aminobutyric acid (GABA)-ergic neuronal populations or specific Purkinje neuronal populations of the cerebellum, demonstrate earlier onset of the disease phenotype with prominent motor disturbances and dramatically shortened life span. The reasons underlying predilection of certain brain regions, such as the brainstem and cerebellum, in global transgenic models are not fully understood. While human post-mortem tissues represent the most valuable source for mechanistic investigation of human mitochondrial disease, they typically exhibit end-stage neuropathology and, therefore, do not provide an insight into the temporal disease dynamics, for which mouse models are required. Current mouse models have limitations and do not fully recapitulate the complexity of human disease phenotypes; however, the Leigh syndrome *Ndufs4* knockout mouse model seems to exhibit the phenotype and neuropathological lesions in the brain regions relevant to the disease, such as the brainstem. Moreover, we highlight the importance of glial cells’ contribution to the phenotype—for example, astrocytic mitochondrial impairment in establishing spongiotic neuropathology [28] and targeting microglial or leucocyte reactivity by inhibiting their proliferation and differentiation by reducing colony-stimulating factor receptor 1 (CSF1R) signalling as a potential treatment to delay neurological decline in a mouse model of global mitochondrial dysfunction [123].

## Supplementary Materials

The supporting information can be downloaded at: https://www.mdpi.com/article/10.3390/ijms24119698/s1.
